# Adhesive creases: bifurcation, morphology and their (apparent) self-similarity

**DOI:** 10.1039/d2sm01389d

**Published:** 2023-06-30

**Authors:** Martin H. Essink, Michiel A. J. van Limbeek, Anupam Pandey, Stefan Karpitschka, Jacco H. Snoeijer

**Affiliations:** a Physics of Fluids Group, Mesa+ Institute, University of Twente 7500 AE Enschede The Netherlands j.h.snoeijer@utwente.nl; b Max Planck Institute for Dynamics and Self-Organization 37077 Göttingen Germany; c Mechanical & Aerospace Engineering Department and BioInspired Syracuse, Syracuse University Syracuse NY 13244 USA; d Fachbereich Physik, Universität Konstanz, 78457 Konstanz Germany

## Abstract

An elastic material that experiences strong compression parallel to its free surface can exhibit sharp surface folds. Such creases arise due to an instability where a self-contacting fold appears on the surface, often observed in growing tissues or swelling gels. Self-adhesion of the contact is known to affect the bifurcation behavior and morphology of these structures, yet a quantitative description remains elusive. From numerical simulations and an energy analysis we resolve how adhesion quantitatively affects both morphology and bifurcation behavior. It is found that a reduced energy is able to accurately describe the bifurcation, in terms of an effective scaling that collapses the data very well. The model accurately describes how adhesion hinders crease nucleation. Furthermore, we show that the free surface profiles in the presence of surface tension exhibit self-similarity, and can be collapsed onto a universal curve.

## Abstract 2

An elastic material that experiences strong compression can exhibit sharp surface folds, often seen in growing tissues or swelling gels. We resolve how self-adhesion affects both morphology and bifurcation behavior of the adhesive crease.

## Graphical abstract

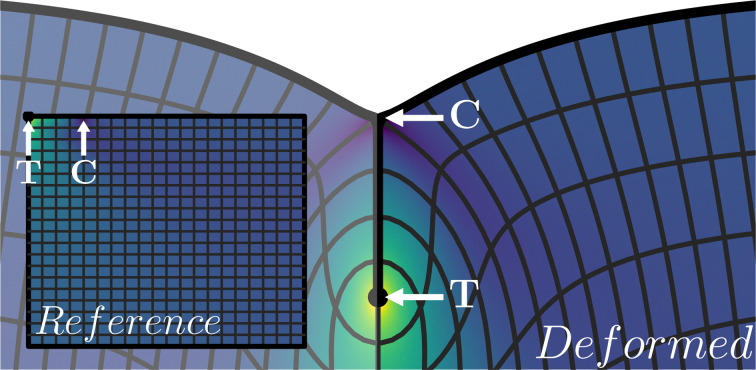

## 1. Introduction

The surface of a block of soft material exhibits intriguing pattern forming instabilities under compressive forces. Creases are a prime example of such surface instabilities where the soft interface folds onto itself to create sharp folds.^1^ These surface features appear in a wide variety of mechanical and natural environments such as swelling or shrinking gels^2,3^ or coatings,^4–7^ bending of elastomers,^8–11^ collapse of cavities within elastic materials,^12^ or growth induced gyrification of mammalian brains^13–16^ and tumors.^2^ Thus the geometry and mechanics of creases have been of significant interest to the scientific community. In the canonical example of compressing a slab of soft, elastic material parallel to its surface, creases nucleate at a critical compressive strain of *ε*_0_ ⋍ 0.35, and their size grows with the strain following a supercritical bifurcation. The mechanism of this is well understood; the crease geometry alleviates strain in the material, decreasing the mechanical energy for a given amount of compression.^17,18^ However, both nucleation of creases upon compression and their disappearance upon relaxation have been found to be highly hysteretic for adhesive substrates. Nucleation is affected by surface defects, which can be strongly dependent on the substrate.^19,20^ Similarly, as the compression is slowly removed, the crease disappears at a strain well below *ε*_0_ as a result of adhesion hysteresis^21^ or contact line pinning.^22^ Experimentally, most of the hysteresis in crease formation can be eliminated by measuring the ‘channeling’ strain, which is the strain required for the crease to propagate on a substrate that increases in thickness perpendicular to the compression. Such a method eliminates the need for nucleation sites, and with that the hysteresis in crease formation.^19^

The key feature of a crease is the folded region of self-contact.^10,11,17–20,22–24^ Of particular interest is the case where this contact represents a gain in surface energy, such that strong adhesion is present. This resembles the situation of classical contact mechanics of spherical bodies, where the presence of adhesion fundamentally changes the nature of the contact, from “Hertz” to “JKR” (Johnson–Kendall–Roberts) type, named after the corresponding theory.^25,26^ The nature of the bifurcation from flat to creased states is thus expected to change upon the introduction of adhesion. Indeed, recent experiments have shown that for increasingly soft materials, surface tension drastically modifies the hysteresis loop, and potentially explains the existence of microscopic scars left behind by creases.^19,22^ The competition between surface and elastic energies also alters the micro-morphology of creases near the edge of the self-contact; confocal microscopy experiments point to a transition from Y-shape to T-shape singular morphologies in materials with sufficient softness.^22^ However, a quantitative description of the bifurcation behavior and the morphology of an adhesive crease remains elusive.

In this paper we use finite element analysis to explore the bifurcation behavior and morphology of creases under the effect of surface tension and adhesion. It is found that adhesion indeed changes the nature of the bifurcation, from supercritical to subcritical. Our numerical results are interpreted in detail using a reduced energy, based on an expansion in the crease amplitude. The impact of surface tension turns out to be non-trivial, converging to an asymptotic scaling only very slowly – even though our simulations cover a broader range of typical experimental values. Still, using an apparent local exponent, this reduced energy model exhibits a self-similar structure which, despite approximative in nature, achieves a very good collapse of the numerical bifurcation data over the experimentally relevant range. Furthermore, we find how adhesion alters the morphology of the crease, primarily near the edge of the self-contact. The crease morphology exhibits self-similarity, with a proper scale-invariance, by which we quantify the effect of surface tension on the shape of the free surface in the vicinity of the self-contact.

## 2. Methodology

### 2.1 The elastocapillary free energy

**Fig. 1:**
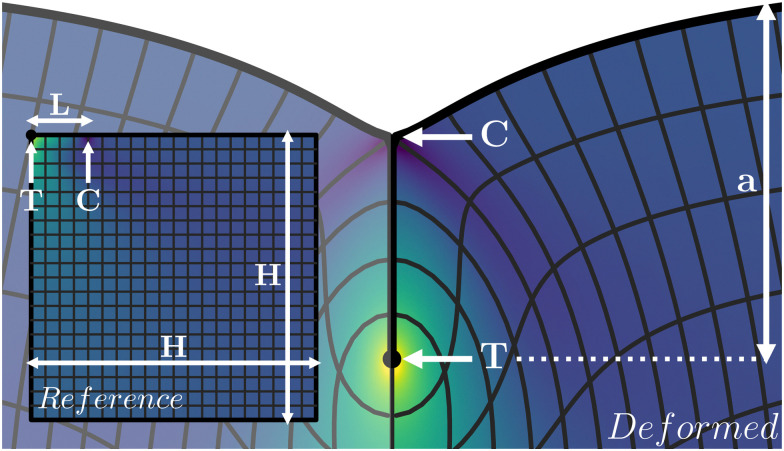
Schematic representation of a crease, with the relevant length scales and points indicated in white. Indicated are the crease amplitude *a*, the tip of the crease T and the contact line C. The mesh represents an evenly spaced grid in the reference configuration (inset, only right half shown), and the colormap indicates the solid pressure.

We study creases in the presence of surface tension and adhesion by considering the lateral compression of a slab of elastic material. The approach closely follows that of,^18,23^ with the addition of surface tension.^22^ Elastic deformations are characterized by the mapping **x** = *f*(**X**), which deforms the coordinates in the reference configuration **X** to those in the current configuration **x** (*cf.*Fig. 1). Equilibrium states are obtained numerically through minimization of the total free energy, which consists of elastic and capillary contributions. The volumetric density of elastic energy *W*(**F**) is a function of the deformation gradient tensor **F** = ∂**x**/∂**X**. Specifically, we will consider an incompressible Neo–Hookean solid. In plane-strain conditions, the Neo–Hookean energy density per unit length in the out-of plane direction reads1
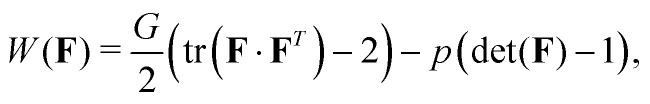
were we introduced the shear modulus *G*, while the constraint of incompressibility is included *via* the Lagrange multiplier *p*. The total elastic energy is obtained by integrating *W* over the reference domain. Further, we introduce a surface energy *γ*, which contributes everywhere along the free surface of the medium. Inside the self-contact, we assume a perfect adhesion so that this region does not contribute to the surface energy. Outside the self-contact, on the free surface, we consider *γ* to be constant (*i.e.* we do not include the possibility of surface elasticity/Shuttleworth effect). Note that *γ* represents an Eulerian energy density, *i.e.* it is measured per unit area in the current configuration.

We now describe the geometry in more detail (*cf.*Fig. 1). In what follows, all lengths measured in the reference state (Lagrangian) are denoted by capitalised symbols, while lengths in the deformed configuration (Eulerian) are lowercase. We consider a square domain of size *H* × *H*, subject to plane strain conditions, which is compressed horizontally from the sides to Eulerian width *w*. The global compression is defined as

2 *ε* = 1 − *w*/*H*.

The self-contact of Lagrangian length *L* is located at the top-left corner of the domain, from fold line T to contact line C. Since the crease is symmetric, the numerical domain only contains the right half of the crease. All interfaces, but the free surface, of the elastic slab are subject to symmetry conditions, including the self contact. Effectively, this corresponds to an elastic layer of thickness 2*H* with creases at 2*H* intervals on the top and bottom. Now that the domain is specified, one can make explicit the total mechanical energy of a crease structure, per unit length in the out-of-plane direction. Using the symmetry *X* → −*X*, this gives3
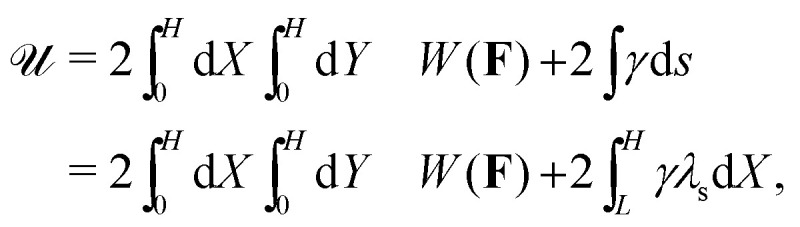
where *λ*_s_ is the surface measure in the current configuration. The functional 𝒰 is minimized with respect to the mapping *x* = *f*(**X**), under constraints that represent the boundary conditions described above.

### 2.2 Control parameters and non-dimensionalisation

In an experiment, one would typically impose the global compression *ε* and measure, *e.g.*, the resulting crease amplitude *a*, defined as the vertical Eulerian distance between T and the flat free surface. In the numerical scheme, however, the self-contact is explicitly imposed, by enforcing a self-contact of fixed Lagrangian length *L* along the surface, from point T to point C (Fig. 1). The global compression *ε* and the deformation field, including the Eulerian crease length and the deformation amplitude *a*, are then found self-consistently from energy minimization.

To quantify the balance between surface and elastic contributions to the total energy, we define the elastocapillary length^27,28^

4 *

<svg xmlns="http://www.w3.org/2000/svg" version="1.0" width="13.454545pt" height="16.000000pt" viewBox="0 0 13.454545 16.000000" preserveAspectRatio="xMidYMid meet"><metadata>
Created by potrace 1.16, written by Peter Selinger 2001-2019
</metadata><g transform="translate(1.000000,15.000000) scale(0.015909,-0.015909)" fill="currentColor" stroke="none"><path d="M480 840 l0 -40 -40 0 -40 0 0 -40 0 -40 -40 0 -40 0 0 -120 0 -120 -80 0 -80 0 0 -40 0 -40 40 0 40 0 0 -80 0 -80 -40 0 -40 0 0 -80 0 -80 40 0 40 0 0 -40 0 -40 80 0 80 0 0 40 0 40 40 0 40 0 0 40 0 40 -40 0 -40 0 0 -40 0 -40 -40 0 -40 0 0 160 0 160 40 0 40 0 0 40 0 40 40 0 40 0 0 40 0 40 40 0 40 0 0 40 0 40 40 0 40 0 0 80 0 80 -40 0 -40 0 0 40 0 40 -40 0 -40 0 0 -40z m80 -120 l0 -80 -40 0 -40 0 0 -40 0 -40 -40 0 -40 0 0 80 0 80 40 0 40 0 0 40 0 40 40 0 40 0 0 -80z"/></g></svg>

*_ec_ ≡ *γ*/*G*.

This length describes the scale below which capillarity starts to dominate over elasticity. Since the adhesion within the self-contact is considered to be ideal, the local force balance at C leads to a 90° contact angle between the free surface and the self-contact once **_ec_ > 0.^22^ This results in the distinctive T-shape at the edge of the self-contact, as is visible in Fig. 1. The problem is then defined by three length scales: the layer thickness *H*, the length of the self-contact *L*, and the elastocapillary length **_ec_. Hence, the dimensionless problem contains two control parameters, which we choose to be *L*/*H* and **_ec_/*H*.

In practice, we are interested in cases where the crease is small compared to the layer thickness, which implies both *L*/*H* and *a*/*H* to be small. In addition, experimental values of *ℓ*_ec_ are typically smaller than the crease size. Hence, in the results below we have5
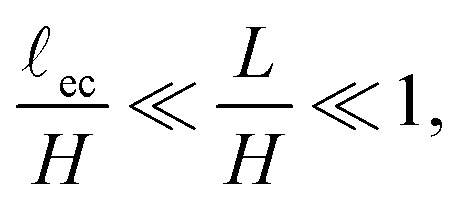
which reflects the typical experimental hierarchy of scales for thick layers, close to the creasing threshold.

### 2.3 Numerical implementation

**Fig. 2:**
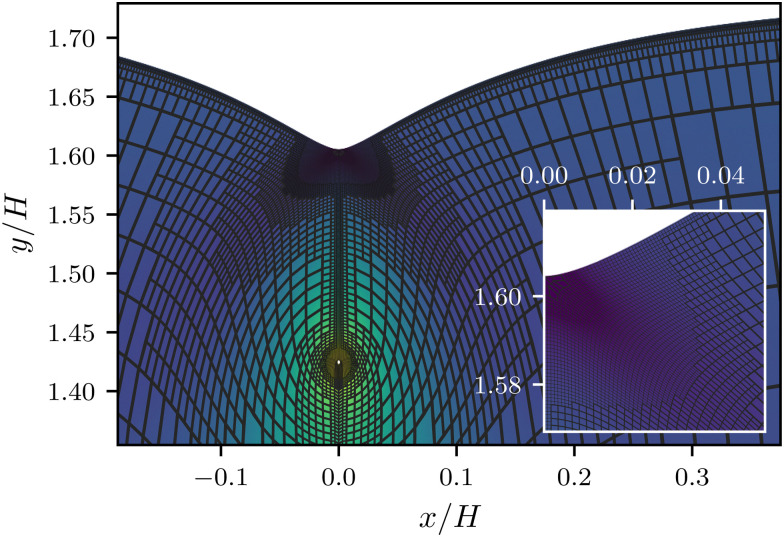
A typical numerical result, including the full refined mesh. For visual clarity a large self-contact *L*/*H*= 0.2 and elastocapillary length *γ*/*GH* = 0.016 are used.

The numerical results presented in this manuscript are generated by the finite element method (FEM) simulation library oomph-lib.^29^ The simulation methods are based on those in previous work,^18^ but now extended to include capillary and adhesive effects. A schematic overview of the deformed configuration with key length scales is shown in Fig. 1. The bulk of the material is simulated using a mesh (*H* × *H*) of Neo–Hookean solid quad elements, refined in a fixed predefined pattern. The most refined elements (*Δ* = 9.8 × 10^−4^*L*) are located at the free surface and along the length of the fold, while the least refined elements (*Δ* = 0.25*L*) are found at the bottom right. Typically, 10*L* ≲ *H* ≲ 100*L*. Fig. 2 shows a typical example of a solution with the refinement pattern. Further details on the numerical implementation can be found in the Appendix.

## 3 Bifurcation behaviour

### 3.1 Crease amplitude

**Fig. 3:**
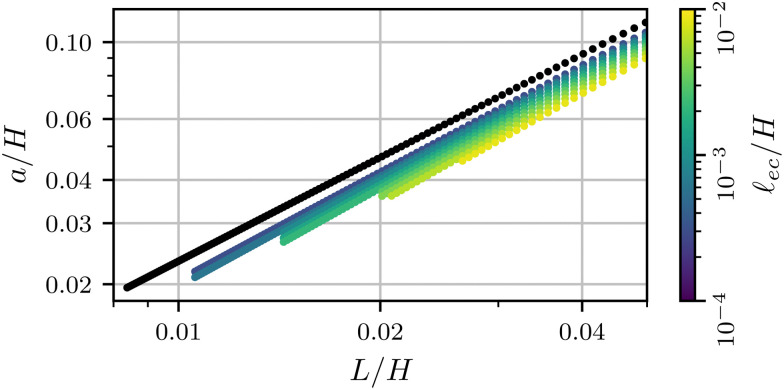
The size of the crease *a* as a function of the contact length *L* for various values of the elastocapillary length. The data is colored according to *ℓ*_ec_/*H*, with the non-adhesive data shown in black.

To interpret the relations between input and output quantities, we start by plotting the crease amplitude *a*/*H* as a function of the self-contact length *L*/*H*, as shown in Fig. 3. The black symbols correspond to the case without surface tension, *ℓ*_ec_/*H* = 0, while the colored symbols represent data with increasing effect of capillarity. In all cases we observe a nearly linear relation between the (Lagrangian) contact length and the (Eulerian) crease amplitude, showing that both are nearly equivalent measures to quantify the size of the crease. One notices, however, that the crease amplitude is decreased for increasing *ℓ*_ec_/*H*: the effect of surface tension is to flatten the surface outside the self-contact.

**Fig. 4:**
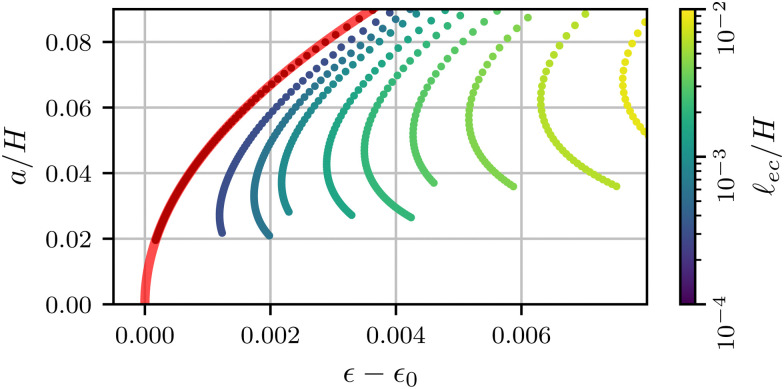
Numerical bifurcation diagrams for the crease size *a* as a function of compression *ε*. The black dots indicate the profile for the special case *Γ* = 0. A fit of the theoretical prediction (8) is shown as a translucent red line.

We now turn to the creasing bifurcation diagrams, and discuss how these are affected by capillary effects. Fig. 4 presents a parametric plot, showing the crease amplitude *a*/*H* as a function of the imposed strain *ε*. Different colors represent different values of the *ℓ*_ec_/*H*. The non-adhesive case (*ℓ*_ec_/*H* = 0) is shown as the black symbols, overlaid by the red curve that is a perfect fit with a square-root behavior 
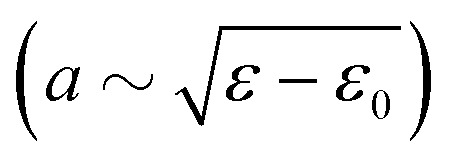
. This fit shows that the non-adhesive crease exhibits supercritical bifurcation, where the crease appears at a critical value *ε*_0_. We numerically find *ε*_0_ ⋍ 0.352, in perfect agreement with previous simulations.^20,23^

However, when the elastocapillary length *ℓ*_ec_/*H* becomes nonzero, the bifurcation behavior changes fundamentally. The minimal compression for crease formation increases with *ℓ*_ec_/*H*, and the size of the crease becomes nonzero at the critical point. This shows that the introduction of surface tension leads to a transition from a supercritical to a subcritical bifurcation. The fact that surface tension induces a finite crease-amplitude at the critical point was previously anticipated^19^ and confirmed in numerical simulations.^20^ The purpose of the present study is to identify the exact bifurcation structure near the critical point; specifically, we aim to identify the relevant scaling laws that characterise the crease formation in the presence of surface tension.

### 3.2 Interpretation: the reduced elastocapillary energy

To interpret the bifurcation results we reduce the fully resolved energy through an expansion in the crease amplitude *a*. This approach offers a simplified form of the free energy, where the complete functional^3^ is reduced to an algebraic expansion in *a*. Such an analysis is inspired by the approach taken in;^19,20^ we will here see how the appropriate scaling can approximate the data in Fig. 4 by a universal bifurcation curve.

For notational convenience, we make use of non-dimensional quantities in the following:6
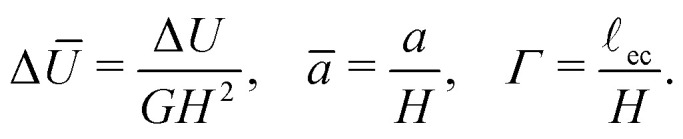
Here, Δ*U* is the difference in energy per unit length *U* between a creased state and a state of uniform compression with the same *ε*. The effect of surface tension is encoded in *Γ*, which, given the hierarchy of length scales^5^ can be considered small here.

#### 3.2.1 Non-adhesive creases

We start the discussion with the non-adhesive crease, *i.e. Γ* = 0. In the vicinity of the crease tip *T*, the elastic energy density is uniform and dictated by the folding geometry.^30^ Given that the crease is generated above *ε*_0_, we anticipate the reduction of energy (with respect to the uniform compression) is proportional to Δ*εā*^2^, where we define Δ*ε* = *ε* − *ε*_0_. In order to reach a saturation of the crease amplitude, a higher order term ∼*ā*^*α*^ with *α* > 2 must be included. We thus write the expansion

7 Δ*Ū*_0_ = −*c*_2_Δ*εā*^2^ + *c*_*α*_*ā*^*α*^.

The subscript 0 of the energy indicates that *Γ* = 0, while the exponent *α* remains to be identified.

The form (7) was previously suggested,^19,24^ with a value *α* = 3. This value, however, is not consistent with the supercritical bifurcation observed in Fig. 4. Namely, for the equilibrium states obtained from the energetic minima, ∂Δ*Ū*_0_/∂*ā* = 0. Solving this expression for Δ*ε* yields8
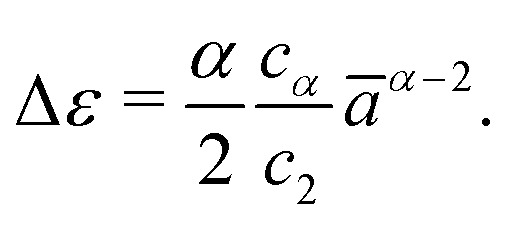


For *α* = 3 one obtains a linear relation between *a* and Δ*ε*, while the numerical results clearly exhibit a square-root behavior. Indeed, the red curve fitted in Fig. 4 corresponds to (8) with *α* = 4. It is somewhat unexpected that the expansion misses a cubic term, which suggests that the elastic energy admits only even powers of *ā*. Phenomenologically, the appropriate expansion parameter for the elastic energy appears to be the characteristic area of elastic deformation *ā*^2^, rather than the crease amplitude. This observation can perhaps be rationalized from the fact that a negative value for *ā* does not have any physical meaning; this lack of *ā* → −*ā* symmetry could therefore be the underlying reason why *ā*^2^ is the correct expansion parameter for the elastic energy. The absence of a cubic term furthermore implies that previously proposed scaling arguments need to be revised, as will be discussed below.

As another route to explicitly verify the value of the regularisation exponent *α*, we evaluate the energy of the crease, by inserting the equilibrium value for *ε*. This gives

9 Δ*Ū*_0_|_eq_ = −*c*_4_*ā*^4^.

**Fig. 5:**
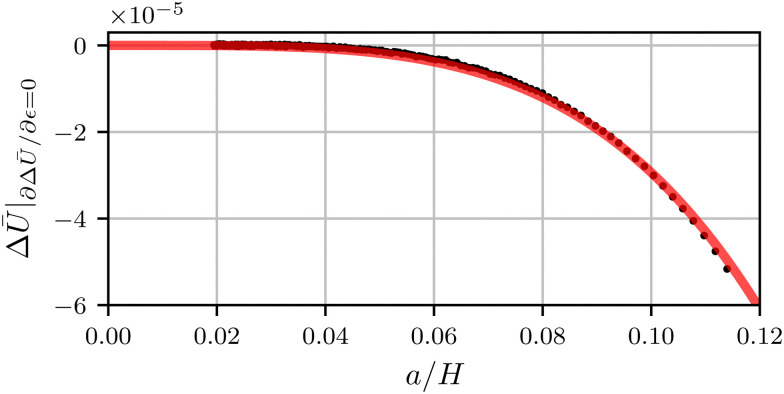
The energy difference between a creased and flat state as a function of the crease size. A fit of the theoretical expression (9) is shown as a translucent red line.

This prediction can be compared directly to the exact expression of the full elastic energy obtained from the numerical simulations. The result is shown in Fig. 5, where the red curve superimposed is the best fit of (9). The numerical energy indeed follows the quartic behavior, and the prefactor gives the value of *c*_4_. Combined with the fit of the bifurcation diagram, we obtain the coefficients for our configuration, namely, *c*_2_ ⋍ 1.30 and *c*_4_ ⋍ 0.294.

#### 3.2.2 Adhesive creases

Now that the energy of a non-adhesive crease is known, the contribution of surface tension can be added. Given that we work in the limit *Γ* = *ℓ*_ec_/*H* ≪ 1, we will assume that the effect of surface tension can be added perturbatively, such that the numerical coefficients of the elastic energy (*ε*_0_, *c*_2_, *c*_4_) are unaffected. In dimensional units, the additional energy (per unit depth) due to capillarity is given by *γ*Δ*ℓ*_s_, where Δ*ℓ*_s_ is the added length of the free surface with respect to the flat state. When the effect of surface tension is very small, the crease morphology can be estimated from the non-adhesive crease, for which the numerics give Δ*ℓ*_s_ = 0.17*a* when *a* ≪ *H*. Hence, in the asymptotic limit *Γ* ≪ 1, the added contribution to the free energy must be of the form Δ*Ū* ∼ *Γā*.

**Fig. 6:**
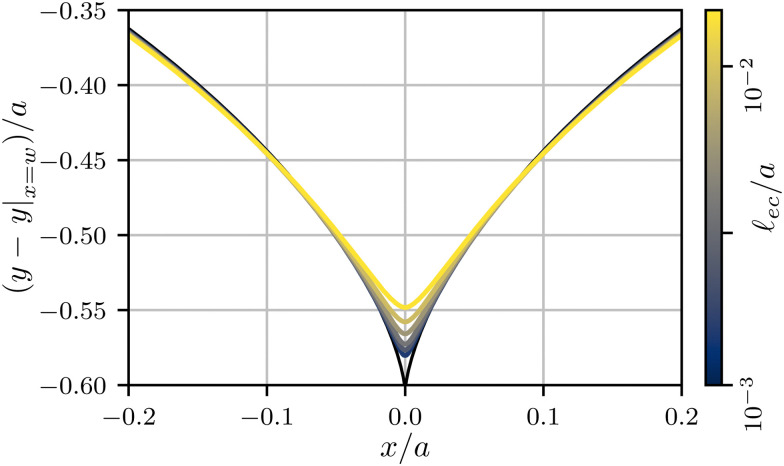
Surface profiles of creases for varying elastocapillary lengths, indicated by the colorbar. The profile of a non-adhesive crease is shown in black. All profiles are aligned by a vertical shift *y*|_*x*=*w*_, the vertical position of the surface far from the crease.

**Fig. 7:**
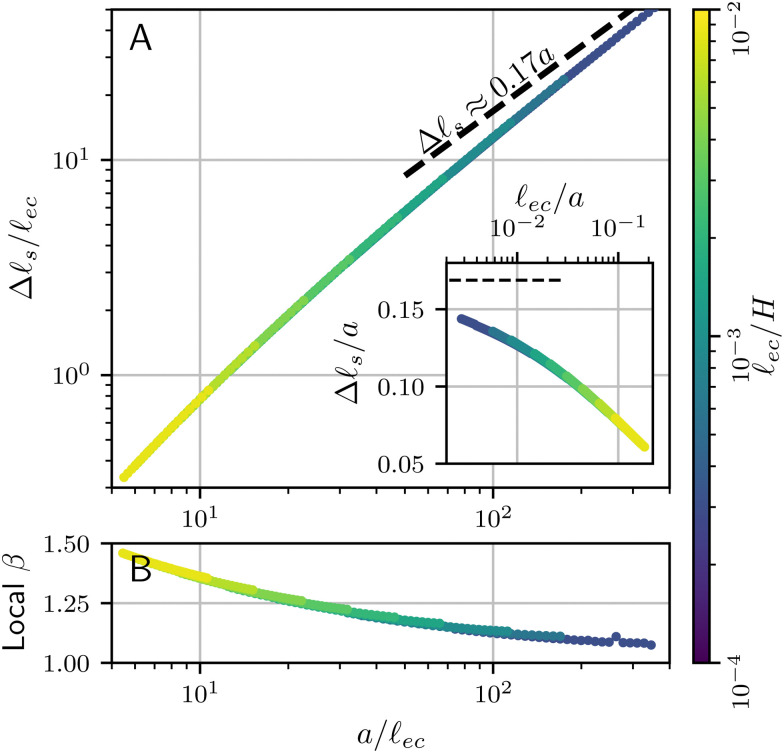
(a) Increase in surface length between a flat and creased state Δ*ℓ*_s_ = *ℓ*_s_ − *w* as a function of the crease size *a*, both normalised by the elastocapillary length *ℓ*_ec_. Also shown are the the asymptotic value obtained from the non-adhesive results (Δ*ℓ*_s_ ≈ 0.17*a*). The inset shows Δ*ℓ*_s_ as a function of the elastocapillary length, normalised by the crease size. (b) The local value of the empirical exponent β, found from fitting the slope of the logarithmic plot in panel a.

However, as can be seen in Fig. 6, the shape of the free surface changes significantly with the introduction of surface tension – even though *Γ* = *ℓ*_ec_/*H* ∼ 10^−4^ and *ℓ*_ec_/*a* ∼ 10^−3^ for the numerical profiles shown here. Hence, the length of the free surface is decreased with respect to the asymptotic result Δ*ℓ*_s_ = 0.17*a*. This reduction of the length is confirmed in Fig. 7(a), where we report Δ*ℓ*_s_ as a function of *a*. The linear asymptote indicated by the dashed line is only very slowly approached, even though the numerics cover a signficant range of very small *Γ*. For this reason, it turns out ineffective to interpret the bifurcation diagram using a linear scaling. Instead, we introduce an effective scaling Δ*ℓ*_s_ ∼ *a*^*β*^, as an empirical representation of the numerical data. Fig. 7(b) shows that the local exponent roughly decreases from 1.5 down to 1.1 as the elastocapillary length decreases from from about 1/5th to about 1/300th of the crease amplitude. Notably, the outer dimension *H* has no significant impact on the crease morphology: Data for various *H* (from yellow to dark blue) overlap almost perfectly. While this slowly approaches the true asymptotic value *β* = 1, an empirical *β* ≈ 1.2 offers a much better approximation over the broad range of numerical data. For this reason, we introduce the effective exponent *β* in the energy contribution of the surface tension, and propose

10 Δ*Ū* = *c*_*β*_*Γ*^2−*β*^*ā*^*β*^ − *c*_2_Δ*εā*^2^ + *c*_4_*ā*^4^.

In this expression we used as a closure the assumption that *H* does not appear in the capillary term, which is achieved by the scaling with *Γ*^2−*β*^. While the true small *Γ* asymptotics will be given by *β* = 1, we will find that, in practice, a much better description of the numerical results is indeed obtained for *β* = 1.2. The values for *ε*_0_, *c*_2_ and *c*_4_ are kept at the same value as the non-adhesive crease.

### 3.3 Apparent scaling and apparent self-similarity of the bifurcation

Once again, a bifurcation scenario can be derived by minimisation of (10) with respect to *ā*. It is instructive, however, to first proceed by introducing a scaling that eliminates *Γ* from the energy. Even though true universality and scale-invariance is not implied outside the asymptotic scaling regime, the resulting bifurcation curve serves as a good approximation over the wide range of numerical data.

We propose an empirical, approximative self-similar structure by introducing a scaling of the form,

11 Δ*ε̂* = *Γ*^−*η*^Δ*ε*, *â* = *Γ*^−*ζ*^*ā*, Δ*Û* = *Γ*^−*χ*^Δ*Ū*.

Substituting this into (10) yields the scaled expression for the energy difference.

12 *Γ*^*χ*^Δ*Û* = *c*_*β*_*Γ*^2−*β*+*βζ*^*â*^*β*^ − *c*_2_*Γ*^*η*+2*ζ*^Δ*ε̂â*^2^ + *c*_4_*Γ*^4*ζ*^*â*^4^.

To find a *Γ*-independent solution, each of the exponents of *Γ* in (12) should be equal: *χ* = 4*ζ* = *η* + 2*ζ* = 2 − *β* + *βζ*. Solving this system results in values for the three unknown exponents,13
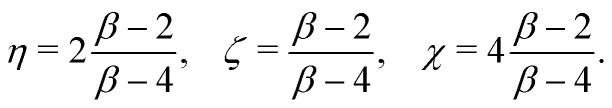
Under this scaling law the universal profile of the bifurcation diagram is,14
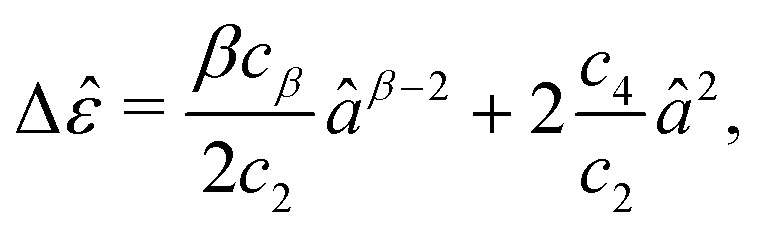
containing the exponent *β* and the coefficient *c*_*β*_ as unknown parameters. Additionally, substituting this solution into (12) yields the energy difference along the bifurcation curve,15
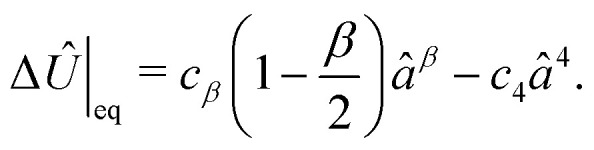
Indeed, the scaled expressions for Δ*ε̂* and Δ*Û* are independent of *Γ* and should yield a decent collapse of the numerical bifurcation curves and the corresponding energy.

**Fig. 8:**
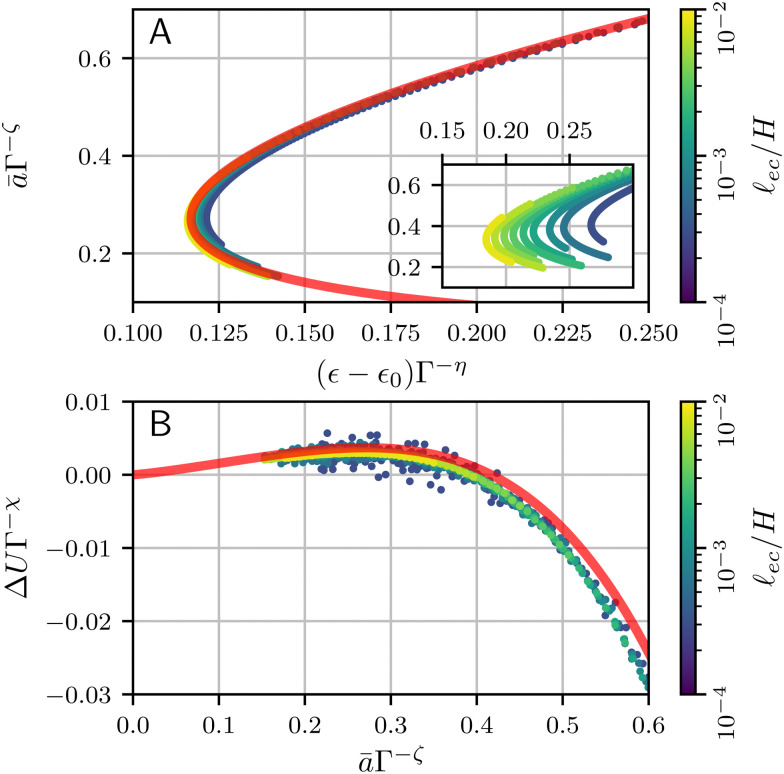
(A) Numerical bifurcation diagrams for several values of *Γ*, approximately collapsing onto (14) (red line) using the approximate scaling (11). Inset is the same figure, scaled with *β* = 1. (B) Energy difference between the creased and flat state, collapsed onto the universal curve (15). Parameter values (*c*_*β*,2,4_ and *β*) are calculated from fits to numerical profiles, and are provided in the main text. The numerical profiles correspond to those shown in Fig. 4.

To test this scenario, we first recall that the true asymptotics for *Γ* → 0 has *β* = 1, for which the exponents become16
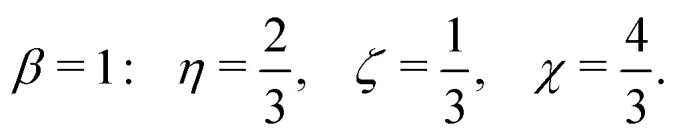
The inset of the Fig. 8(a), plotting *â versus* Δ*ε̂* shows that this scaling groups the data, but does not really lead to a collapse. Given the relatively small value of *Γ*, this implies that the true asymptotics is only very slowly approached. As anticipated, an empirical, effective value of *β* = 1.2 gives a better estimate of the capillary energy, in which case the scaling exponents become

17 *β* = 1.2: *η* = 0.57, *ζ* = 0.29, *χ* = 1.14.

Indeed, the main panel of Fig. 8(a) shows that these exponents lead to an excellent collapse of the data. The red curve corresponds to (14), with a fitted coefficient *c*_*β*_ = 6.39 × 10^−2^. We also verified that the scaling works for the crease energy Δ*Û*|_eq_., reported in Fig. 8(b). Once again, a collapse is obtained and the red line provides (15) without any adjustable parameters, taking into account that the value of *β* follows empirically from Δ*ℓ*_s_*vs. a*.

### 3.4 Experimental implications

A few closing remarks in relation to experiments are in order. First, we note that a crease is only energetically favourable for *â* = *āΓ*^*−ζ*^ ≳ 0.4, as can be seen in Fig. 8(b); only for those values the energy difference with respect to the flat state becomes negative. Inspecting Fig. 8(a), one observes that *â* = 0.4 does not coincide with the minimum value of Δ*ε* along the curve; hence, only part of the upper branch will lead to a global minimum. From an experimental perspective, such a scenario was already proposed in the discussion of “channeling” of creases.^19^ In these experiments a wedge-shaped substrate is compressed such that creases form on the thinnest part and propagate, or channel, towards the thicker region. This eliminates the need for nucleation sites, eliminating most hysteresis of crease formation. Channeling was found to require an “overstrain” with respect to *ε*_0_ (the critical strain for non-adhesive creases). A scaling law for the overstrain was experimentally found^19^ to scale as Δ*ε* ∼ *Γ*^*η*^, with *η* = 0.49 ± 0.06. This result was interpreted using a cubic regularisation term in the elastic energy and *β* = 1, which gives *η* = 1/2. While this is close to the observed exponent, we now know that the regularizion involves a quartic elastic term. Therefore, we propose an alternative interpretation: Owing to the slow approach of the asymptotic crease shape, the quartic term in combination with an emperical scaling *β* = 1.2 gives the value *η* = 0.57. This is close to the experimental observation, in particular when noting that the experimental observations are at a comparatively large *ℓ*_ec_/*a*, which fall in the range ∼0.1⋯10. Based on Fig. 7, this would give an even further increase in the effective value *β*, which brings the exponent *η* even further towards the experimentally observed value. Thus, an important conclusion of our work is that even though the true asymptote *β* = 1 should exist for *a*⋙*ℓ*_ec_, it is not expected to be reached in the experiments.

## 4. Crease morphology

We now wish to discuss the effect of surface tension on the morphology of the crease. We have already seen in Fig. 6 that the crease amplitude is decreased by the influence of surface tension. The fine structure of the crease can be characterized in much more detail. Unlike the bifurcation diagram, which involves large scale features of the interface, our numerics suggest the small-scale features of the crease exhibit a truly self-similar structure. The analysis below closely follows previous work,^22^ which is here complemented by additional numerical data and a new rescaling for the curvature profiles.

### 4.1 Inner and outer profiles

**Fig. 9:**
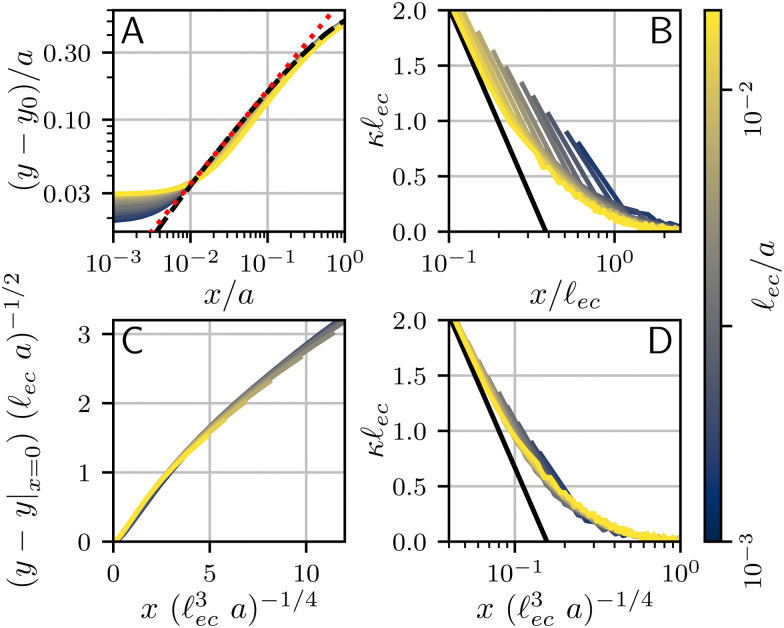
Numerical surface profiles of adhesive creases, for a number of elastocapillary lengths. For each profile the contact length was kept constant at *H*/*L* = 10. (a) Surface height *y* in the outer region (*ℓ*_ec_ ≪ *x* ≪ *a*), scaled with the crease size *a*. The vertical offset of the numerical solutions is determined by the axis-intersection of a fit to eqn (18), plotted as a red dotted line. The profile of a non-adhesive crease is plotted in a dashed black line. (b) Surface curvature 
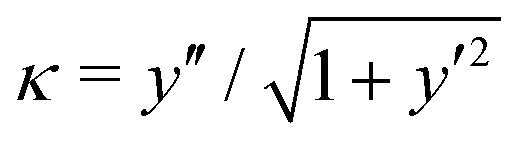
 in the capillary dominated region (*x* ≪ *ℓ*_ec_), scaled with the elastocapillary length *ℓ*_ec_. The black line shows the theoretical prefactor from (19). (c) Surface height profiles collapsed onto a single, universal curve by scaling (23), offset by the surface height at the edge of the self-contact. Since the universal scaling only holds for *x* ≪ *a*, only the range *x* < 0.2*a* is shown, approximately corresponding with the deviation from (18) in (b). (d) Similar to (b), but scaled according to eqn (20).

We first focus on the large-scale features of the crease. For this we plot the free surface profiles scaled by the crease amplitude *a*, in Fig. 6 and, on logarithmic scales, in Fig. 9(a). The profiles for various elastocapillary lengths are seen to collapse nicely on the large scale, where they approach the shape of a non-adhesive crease (superimposed as the black-dashed line). This shows that, as far as the large-scale features of the interface are concerned, the crease amplitude *a* offers the relevant length scale. We further remark that creases without surface tension exhibit the typical cusp scaling,^18^18
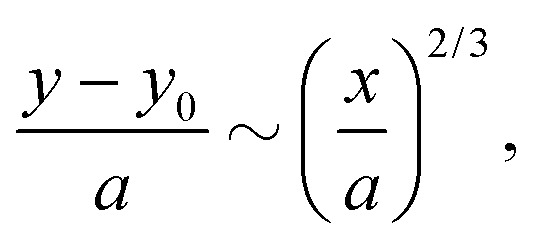
which is shown as the red dotted line; it overlays perfectly with the non-adhesive crease at small distances (black dashed). However, it is clear that this behavior is no longer followed for the profiles with surface tension; in addition, the curves for various *ℓ*_ec_/*a* no longer collapse at small distances *x*. This departure from the non-adhesive crease, and the subsequent breakdown of (18), must obviously be attributed to surface tension.

We now turn to the central region, where capillary effects are expected to be dominant. As can be seen in Fig. 2, the elastic medium at *x* = 0 near the free surface is folded into a sharp 90° contact angle. The local mechanics near this fold is thus expected to approach a folded elastic halfspace, which has been solved analytically.^30^ Specifically, the fold solution is described by an internal Lagrangian angle *Θ* that is folded to and Eulerian angle *θ*. For a neo-Hookean solid, the fold mechanics gives rise to a pressure distribution.19
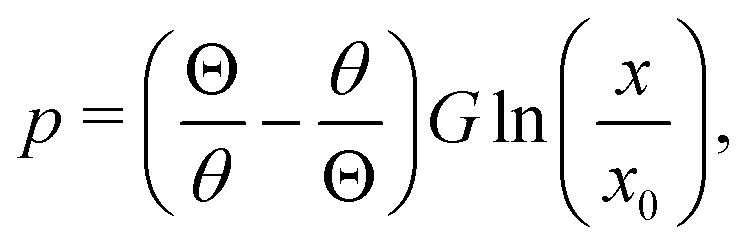
where *x*_0_ reflects an integration constant that is inherited from larger scales. Using Laplace's law of capillarity, we can now directly relate this pressure singularity to the interface curvature *κ*, according to *p* = −*γκ*. Using that *θ*/*Θ* = 1/2 for a fold of 90°, we thus expect the curvature to scale as20
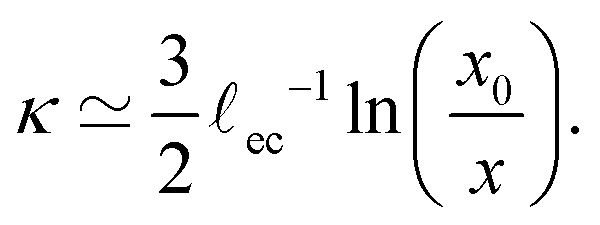
This logarithmic divergence of curvature is indeed observed in Fig. 9(b), where we scaled both *κ* and *x* with the elastocapillary length *ℓ*_ec_. The black line provides the scaling (20), showing a very good match of the data – in particular of the prefactor 3/2 in (20). However, we also observe a horizontal offset between the various curves in Fig. 9(b). This suggests that the horizontal inner scale *x*_0_ is not provided by *ℓ*_ec_, as was previously assumed.^22^

### 4.2 Self-similarity

We have identified the scaling forms for the outer and inner shapes of the crease, respectively given by (18) and (20). However, adhesive creases exhibit a self-similar structure that covers all relevant scales, from inner to outer, provided that *x* and *y* are both small compared to *H*. This self-similar nature can be revealed by normalising *x* and *y* by unknown scales *ℓ*_*x*_ and *ℓ*_*y*_, such that *X̄* = *x*/*ℓ*_*x*_ and *Ȳ* = *y*/*ℓ*_*y*_. Inserted in (18) and (20), this gives

21 *ℓ*_*y*_*Ȳ* ∼ *a*^1/3^*ℓ*_*x*_^2/3^*X̄*^2/3^,

22
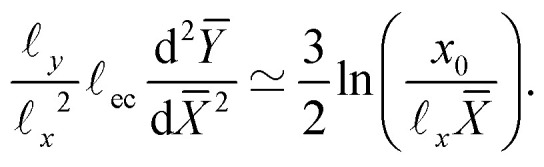
Self-similarity implies that both expressions be universal in the sense that they do not depend explicitly on *a* nor on *ℓ*_ec_. Such an independence can indeed be achieved, by choosing the horizontal and vertical scales as

23 *ℓ*_*x*_ = *ℓ*_ec_^3/4^*a*^1/4^, *ℓ*_*y*_ = *ℓ*_ec_^1/2^*a*^1/2^.

With this, *a* and *ℓ*_ec_ now drop out and we find

24 *Ȳ* ∼ *X̄*^2/3^,

25
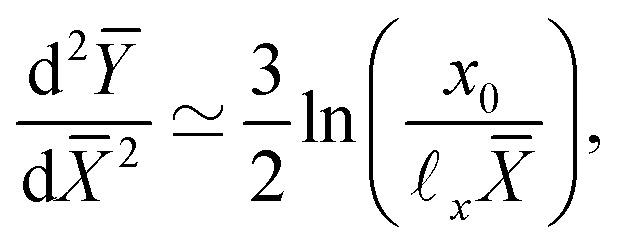
which are devoid of any parameters; apart from the unknown *x*_0_.

Rescaling the numerical crease shapes using *ℓ*_*x*_ and *ℓ*_*y*_, one indeed simultaneously collapses the data of Fig. 9(a and b), now shown in Fig. 9(c and d). Both representations, the curvature and the spatial profiles, reveal the universal structure of creases. The adhesive creases are thus indeed self-similar, with characteristic scales described by (23). The collapse also implies that *x*_0_ appearing in (25) scales like *ℓ*_*x*_. The black line in Fig. 9(d) corresponds to a fitted value *x*_0_/*ℓ*_*x*_ = 0.16.

## 5. Conclusion

In this paper, we have explored the effect of capillary forces on the bifurcation behavior and the morphology of creasing. In accordance with previous work, we have found that surface tension on the free surface and adhesion within the self-contact oppose the formation and growth of creases. In particular, the supercritical bifurcation behavior of a non-adhesive crease becomes subcritical, leading to the hysteresis observed in experiments. By introducing a reduced free energy (10), the bifurcation profiles for a range of elastocapillary lengths can be collapsed. The form of the reduced energy differs from that proposed previously in the literature, but is based on full numerical simulations of creases and in line with experiments on the channeling of creases.^19^ We remark that only for the morphology a true self-similar structure is found. While the bifurcation curves look alike, and can be collapsed very well onto a universal curve, these are not self-similar in a strict sense since a slowly varying empirical exponent remains over the range explored by our numerical simulations.

It is of interest to compare these findings to our previous experimental results.^22^ Specifically, we can compare the bifurcation diagram of the crease amplitude with the compression, with that observed experimentally. These experiments showed a significant history-dependence for a growing or shrinking crease, which was attributed to contact line pinning. Such pinning is typically associated to defects on the surface. Experiments also reveal two distinctive crease morphologies for compression and release, which suggests pinning forces that give rise to differences between advancing and receding contact lines. Regardless of this difference, the advancing and receding morphologies can be collapsed independently using the scaling arguments presented here. Having said that, the pinning behavior is clearly not captured in our numerical simulations: the simulations are based on a perfectly homogeneous substrate for which no pinning to specific material points can occur. Since most differences between experiments and simulations appear driven by contact line pinning, adding it to the numerical method would be an interesting avenue for future work.

## Conflicts of interest

There are no conflicts to declare.

## Supplementary Material

## Figures and Tables

**Fig. 10:**
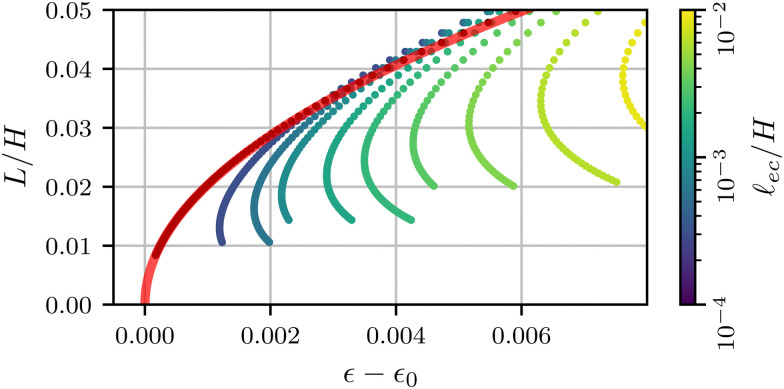
Numerical bifurcation diagrams for the contact length *L* as a function of compression *ε*. The black dots indicate the profile for the special case *Γ* = 0. A fit of the theoretical prediction (8) is shown as a opaque red line.

## Appendices

### Numerical details

The interfaces of the slab are divided into five distinct regions: fold, free surface, and right, bottom and left boundaries. The fold (*X* < *L*) and the free surface (*X > L*) together make up the top boundary of the slab in the reference configuration (*Y* = 0). Due to the *x* → −*x* symmetry of the crease, the fold and the left side of the slab are horizontally constrained at *x* = 0. The right side is constrained at *x* = *w* < *H*, imposing the compression, and the bottom is constrained vertically at *y* = 0. Each of these interfaces is subject to a no-shear condition as well. Lastly, the free surface is subject to a surface energy *γ*. At the bottom of the self-contact a small cutout is made, to avoid the stress singularity of a sharp fold.^18,30^ The top of the self-contact, where it meets the free surface, is a region of interest. The sharp fold at this location will generate a pressure singularity, which we treat by elements that are formulated in polar coordinates, centered around the contact line *C*. The 90° angle is maintained by imposing a vertical force on the node at the contact line. Such a “contact line force” is expected to vanish in the continuum limit. Upon decreasing the mesh size *Δ*, we have verified that this force indeed disappears according to ∼Δ ln(Δ), as expected based on a corner analysis.^27^ Since in our formulation, the contact length is fixed in addition to the thickness and the surface tension, the unknown of the problem is the width of the deformed configuration *w* or the global compression ratio *ε*, which can be found by imposing a local condition near the contact line. From literature^31^ it is known that a fold between two shear free interfaces requires uniform radial and tangential strains. This is achieved by requiring a 45° angle for the line separating the two elements at the contact line (The diagonal line originating from point *C* in Fig. 1).

#### Bifurcation diagram for contact length

In Fig. 10 we show the bifurcation curves similar to the inset of Fig. 8(a), but for the Lagrangian length *L* of the self-contact. These curves show the non-trivial behavior of the length *L*, which unlike the crease size *a* can exceed the length in the non-adhesive situation.
